# Winter cropping in *Ficus tinctoria*: an alternative strategy

**DOI:** 10.1038/srep16496

**Published:** 2015-11-12

**Authors:** Huanhuan Chen, Yanqiong Peng, Yuan Zhang, Richard T. Corlett

**Affiliations:** 1 Center for Integrative Conservation, Xishuangbanna Tropical Botanical Garden, Chinese Academy of Sciences, Menglun, Mengla, Yunnan 666303, China; 2 University of Chinese Academy of Sciences, Beijing 100049, China; 3 Key Laboratory of Tropical Forest Ecology, Xishuangbanna Tropical Botanical Garden, Chinese Academy of Sciences, Kunming 650223, China; 4 Yunnan Academy of Biodiversity, Southwest Forestry University, Kunming 650224, China

## Abstract

The many species of figs (*Ficus*, Moraceae) have evolved a variety of reproductive phenologies that ensure the survival of both the fig plants and their short-lived, species-specific, pollinating wasps. A phenological study of 28 male and 23 female plants of a dioecious hemiepiphytic fig, *Ficus tinctoria*, was conducted in Xishuangbanna, SW China at the northern margin of tropical SE Asia. In contrast to other figs of seasonal climates, which have a winter low in fig production, both sexes produced their major fig crops at the coldest time of the year. Male plants released pollinators during the period when most female trees were receptive and male syconia had a long wasp-producing (D) phase, which ensured high levels of pollination. Female crops ripened at the end of the dry season, when they attracted numerous frugivorous birds and dispersed seeds can germinate with the first reliable rains. Few syconia were produced by either sex during the rest of the year, but these were sufficient to maintain local pollinator populations. We suggest that this unique phenological strategy has evolved to maximize seed dispersal and establishment in this seasonal climate.

The distinctive phenological patterns exhibited by figs (*Ficus* spp.) maintain populations of the tiny, short-lived, species-specific wasps (Hymenoptera: Agaonidae) that are their pollinators^1^,^2^,^3^. Figs have a closed, urn-shaped inflorescence, or syconium, lined with tiny uniovulate flowers. The pollinating wasps enter the syconium through a narrow, bract-covered passage, losing their wings in the process, pollinate the flowers and attempt to oviposit. Ovules that receive a wasp egg form a gall on which the wasp larva feeds, while others if pollinated develop into a seed. The wasps mature and emerge into the fig cavity after a few weeks, with development faster at higher temperatures^4^,^5^. They then emerge from their natal syconium and must find a receptive syconium of the same species within their 1–2 day lifespan^6^,^7^. In most species a suite of non-pollinating fig wasps (NPFW), usually detrimental to the fig’s reproductive success, also raise their offspring in the syconia^8^.

The *ca.* 750 *Ficus* species are distributed throughout the tropics and subtropics^9^ and a variety of reproductive phenologies have evolved in response to differences in the seasonality of rainfall and temperature^2^,^10^. In an obligate mutualism, a successful phenological strategy must work for both partners. The physiologies of the large, long-lived, fig plants and their tiny, short-lived pollinators are expected to respond very differently to climate extremes, with the wasps more sensitive^7^. In agreement with this expectation, pollinating wasps are killed at temperatures a few degrees above current maxima^7^ and go into a reversible coma at low temperatures that do not damage their host plants (unpublished data).

Monoecious figs typically show flowering synchrony at the individual level, which serves to prevent inbreeding, but asynchrony at the population level, which maintains the populations of short-lived pollinators^11^,^12^. The resulting year-round production of ripe syconia makes them important resources for numerous frugivores in the tropics and subtropics^13^, but the climatic sensitivities of the pollinators set a northern limit on the distribution of these taxa^11^,^14^. Phenological continuity at the population level is also essential for the male plants of dioecious species, but the removal of the inbreeding risk allows a wider range of phenological strategies for both sexes. Phenological diversity is particularly noticeable in strongly seasonal climates, where the wasps typically overwinter inside syconia and seasonal peaks of wasp release from male plants are synchronized with the availability of receptive female figs for pollination^2^,^15^,^16^. The reproductive phenology of female plants, in turn, is subject to the same selective pressures as in non-fig species, including the availability of pollinators and seed dispersal agents, and the seasonal occurrence of suitable conditions for seed germination and establishment. These flexible phenological strategies appear to make dioecious fig species better able to adapt to the climatic extremes experienced on the poleward extremes of the range of the genus^2^,^3^,^15^,^17^.

Several different types of phenology have been observed in dioecious fig species^18^. In aseasonal climates, all developmental stages are present year-round at the population level, although male crops are usually synchronized within trees and female crops are synchronized^19^,^20^ or not^21^ in different species. Species in highly seasonal climates, by contrast, tend to produce figs in well-defined crops, with the major annual crop of male figs releasing wasps when the major crop of female figs is receptive, while year-round production of minor male crops ensures the survival of pollinators^15^,^16^. This type of fruiting phenology seems to be frequent in subgenus *Ficus* section *Ficus*^17^,^22^, but is also observed in some species of subgenus *Sycidium* section *Sycidium*^18^ and subgenus *Sycomorus*^3^.

A common feature of the reported phenologies of both monoecious and dioecious fig species in seasonal climates is that pollinator activity occurs largely at the warmer times of the year, presumably reflecting the tropical origin of the fig mutualism. The wasps overwinter as developing larvae in syconia that are produced in summer^16^ or autumn^2^,^3^. Our study area, Xishuangbanna, is located at the northern margin of tropical Asia (21°55′39″N) with a strongly seasonal climate in comparison with most of the tropics (Fig. 1). However, in contrast to other figs of seasonal climates, including other species in the study area, *Ficus tinctoria* shows peak pollinator dispersal in winter. In this study, we made detailed phenology observations in order to understand this apparently anomalous phenological pattern.

## Results

### Syconium and leaf phenology

Syconia were present on most of the *F. tinctoria* trees for much of the year, but there were single, annual crop peaks in both sexes and very few syconia for the rest of the year (Fig. 2). In male trees, the number of syconia began to increase from July, with one branch bearing syconia on each tree, and the peak crop started in mid-October. Female trees reached their crop peaks 1–2 months after male trees. This pattern was repeated in year two and informal observations confirm that it had also occurred in previous years. Some individuals initiated their peak crops out of synchrony with the rest of the population in both years. In male trees, syconia development was slower during the winter peak and the wasp-releasing D phase lasted longer. Wasp emergence from D-phase syconia largely coincided with the availability of receptive B-phase syconia on the sampled trees (Fig. 3). Syconia were not seen on the sampled trees from March to July, but were present on other trees in the vicinity.

*Ficus tinctoria* at XTBG is evergreen (Fig. 4), but leaf replacement was discontinuous and showed no relationship with syconia production in male (GLM: *LR* = 0.17, *P* = 0.68) or female trees (GLM: *LR* = 2.12, *P* = 0.15). Neither minimum temperature nor rainfall were correlated with new leaf initiation in male (GLM: *LR* = 0.58, *LR* = 1.326, *P* = 0.45, *P* = 0.25) or female trees (GLM: *LR* = 0.18, *LR* = 0.25, *P*= 0.67, *P* = 0.62, *P* = 0.29). Fig crop initiation in male trees had no correlation with minimum temperature or rainfall (GLM: *LR* = 1.26, *LR* = 0.40, *P* = 0.26, *P* = 0.53), but in female trees it was positively correlated with minimum temperature (GLM: *LR* = 20.67, *P* < 0.01) but not rainfall (GLM: *LR* = 1.32, *P* = 0.25).

### Reproductive success of *Ficus tinctoria* and its fig wasps

Pollinator production in male syconia was greatest in the crops that preceded the peak crop and lowest after the peak crop (Table 1). Conversely, non-pollinating wasps were lowest in the early crops and highest in the last crop, when they were more abundant than the pollinators.

## Discussion

Although Xishuangbanna is near the northern margins of the Asian tropics the local fig flora is still very rich, with 81 native species: 32 monoecious and 49 dioecious. Despite the highly seasonal climate, the monoecious species that have been studied show the typical phenological pattern of within-tree synchrony coupled with asynchrony at the population level^12^,^23^. The phenology of the dioecious species is more variable, but none of them have a winter peak in crop production^24^,^25^. *F. hispida* trees bear 6–8 asyncronous crops a year with four to five fruit-bearing peaks^26^ and *F. semicordata* trees bear 2–3 synchronous crops a year, with different trees fruiting at different times^27^. Further north in SW China at 31 ^o^N, in a region that experiences winter frosts and some snow, there were syconia on *F. tikoua* males year-round, but very few in winter, with a peak in March-April^3^. Peak wasp release was in late May-June. At a similar latitude in SE China, *F. pumila* has one female crop per year, receptive in spring and maturing in autumn, and two major male crops, with figs receptive in spring and summer^16^. In all dioecious species at the northern limits of fig distribution, the fig wasp populations survive the winter as larvae in slow-maturing syconia on male trees^2^,^3^,^16^. Further south in Thailand at 19 ^o^N, in contrast, most dioecious species produce receptive figs throughout the year, although most wasps are released in the warmer months^28^.

A recent study showed a strong genetic signal in the phenology of individual trees of *F. microcarpa*, raising the possibility that peaks in syconium production at the population level could result from genetic similarity rather than local adaptation^29^. Our study plants, however, were all established naturally from dispersed seeds and form part of a large regional population, so local adaptation is the most likely cause of the observed patterns. Phenological patterns might also be influenced by selection against hybridization between related species that share the same pollinator, as in the *F. auriculata* species group in Xishuangbanna^30^, but *F. tinctoria* is not known to share pollinators with any related species.

The most straightforward explanation for the unique phenology of *Ficus tinctoria* in this study is that it has evolved to maximize female function. In monsoonal tropical Asia, fruiting typically reaches a community-level peak at the end of the dry season and continues into the early wet season^31^. In the northern tropics and subtropics, the fruiting peak is in winter. Fruiting at these times may both enhance seed dispersal, because of a winter influx of fruit-eating birds and diet-switching by omnivorous resident species, and ensure that the seeds have the entire wet season to establish before the onset of the next dry period. This latter advantage may be of particular significance in *F. tinctoria*, which is the only dioecious hemiepiphyte in the local flora. Its seeds must germinate and establish as epiphytes and so establishment is likely to be particularly vulnerable to water stress. The ripe syconia of *F. tinctoria* are small (*ca*. 11 mm diameter) and soft, and thus available to a huge range of frugivorous birds: a study in Xishuangbanna recorded 15 species eating them, with the assemblage dominated by small passerines and barbets^32^. The other hemiepiphytic figs in the region are monoecious and thus unable to show the phenological flexibility of dioecious species.

The male phenology of *F. tinctoria* is also consistent with selection for seed dispersal at the end of the dry season. Male syconia are present year-round in the surrounding area, but there is clearly an early summer bottleneck in wasp production in the study site. The number of syconia and pollinators then increases during later summer so that the peak male crop is occupied in October-November and can produce enough wasps to pollinate the peak female crop in January. This final step requires that the pollinating wasps are active in the coldest period of the year. Fig wasps emerged from male syconia around 08:00–09:00 h. During the peak period from December to January, recorded temperatures in the morning in the study area were 6.3–21.2 ^o^C, with an average of 13.5 ^o^C. Although we have no data for the pollinator of *F. tinctoria*, other local pollinator species have relatively longer lifespans at low temperatures above 10.0 ^o^C and suffer only reversible immobility below this. Moreover, the dry winter weather ensures favorable conditions for wasp flight^28^. *F. tinctoria* reaches its northern limit in Yunnan at LiuKu (25°50′37″N, 98°51′16″E), where minimum temperatures get 5 ^o^C degrees lower in winter. Temperatures below this may make winter pollination impossible.

## Methods

### Species biology

*Ficus tinctoria* subsp. *gibbosa* (Blume) Corner is a hemiepiphytic dioecious fig species belonging to subgenus *Sycidium* section *Palaeomorphe*^33^. Its native range covers tropical and subtropical areas of much of Asia. Plants start as epiphytes, dependent on rainwater and winter fog, but adult trees depend entirely on soil water^34^. The syconia are axillary or just below the leaves, in pairs or solitary. The pollinating wasps are *Kradibia gibbosae*, while non-pollinators recorded from the syconia include *Sycoscapter* sp. and *Philotrypesis ravii* (Sycoryctinae, Pteromalidae), *Neosycophila omeomorpha* (Epichrysomallinae), and *Sycophila* sp. 1, *Sycophila* sp. 2, and *Sycophila* sp. 3 (Eurytomidae).

### Study site

**Figure 1:**
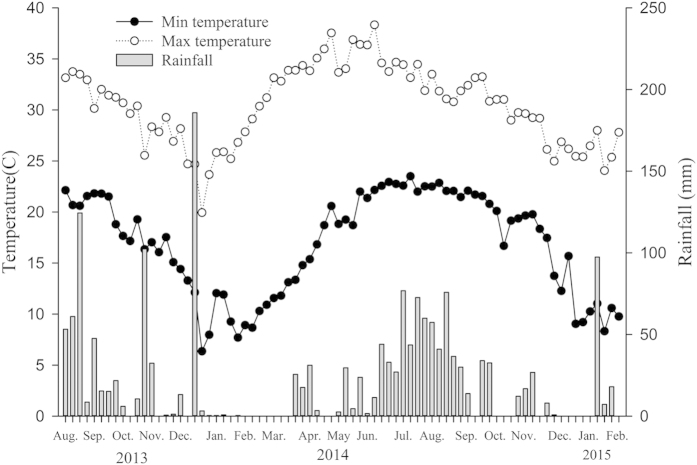
Temperature and rainfall in the study site at XTBG during the period of the study.

**Figure 2:**
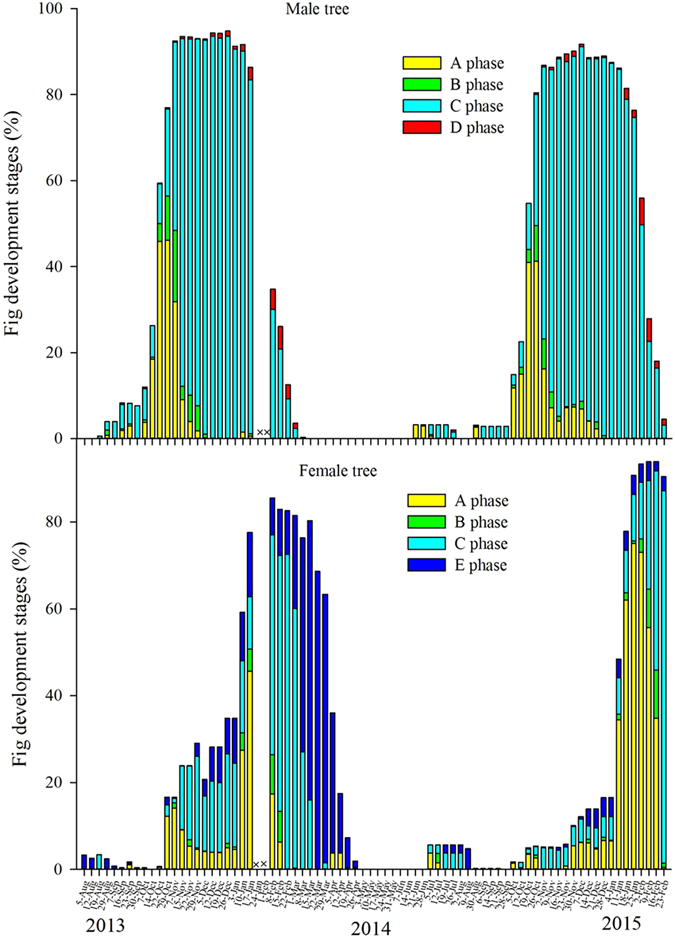
Annual variation in the developmental stages of figs on male trees and female trees (× = missing observations).

**Figure 3:**
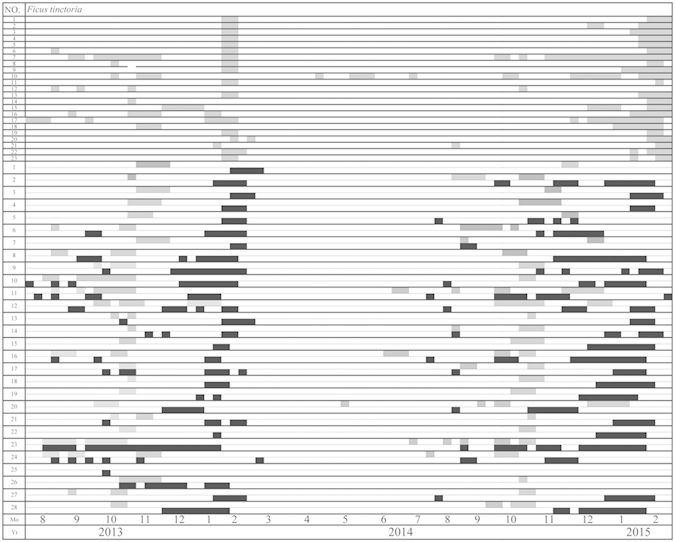
Matching between the production of B phase (grey rectangles) and D phase (black rectangles) syconia. The top 23 plants are female and the bottom 28 male.

**Figure 4:**
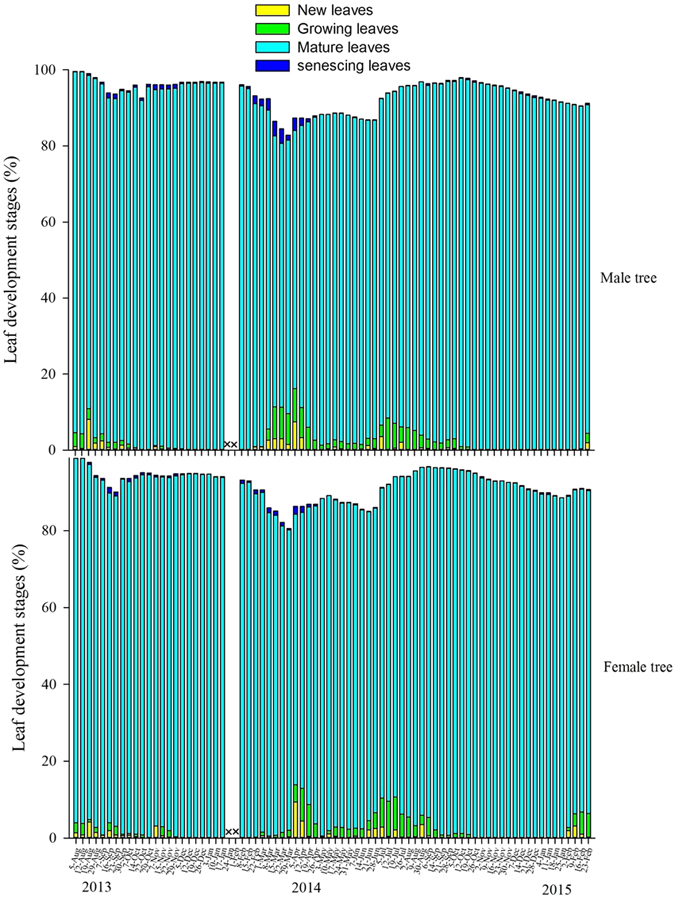
Annual variation in the leaf phenology on male and female trees (× = missing observations).

The study was carried out in and around the Xishuangbanna Tropical Botanical Garden (XTBG) (21°55′39″N, 101°15′40″E), located in SW China at the northern margin of tropical SE Asia. Xishuangbanna has a tropical monsoon climate with strong temperature and rainfall seasonality in comparison with most of the tropics. Annual temperatures (1960–2000) at 558 m asl. average 21.8 °C, with means of 25.7 °C in the hottest month (June) and 16.0 °C in the coldest month (January). The lowest and highest temperatures recorded in the study period were 6.4 °C and 36.3 °C. The mean annual rainfall is 1500 mm. The region experiences three main seasons: a foggy cool season (November to February), a dry hot season (March to April), and a rainy season (May to October). Figure 1 summaries local temperature and rainfall patterns during the period of the study.

### Phenological censuses and data analysis

The 28 male trees and 23 female trees were visited at weekly intervals from August 2013 to January 2015. Individual trees were 10 m to 4 km apart. Most were growing on oil palms, and all were rooted in the ground. The presence or absence of young, growing, mature, and senescing leaves, and the numbers and developmental phases of syconia, were recorded at each visit. Developmental phase classification followed Tzeng *et al.*^35^: pre-female phase (A), female phase (B), interfloral phase (C), male phase (D, on male plants only) and post-floral phase (E, on female plants only). Thirty D-phase syconia per tree were collected. Each syconium was placed individually in a fine-mesh bag (20 × 20 cm) and the fig wasps were allowed to emerge. All the wasps, including those remaining inside the syconia, were collected and preserved in 75% ethanol. The numbers of fig wasps and flowers were counted. Temperature and relative humidity were recorded by Onset HOBO data loggers U23-001 at two hour intervals in three individual trees. Rainfall records from within the study area were provided by the Xishuangbanna Station for Tropical Rain Forest Ecosystem Studies.

The proportions of the trees with new leaves and new syconia were calculated after every census, and related to minimum temperature, maximum temperature and total rainfall during the preceding week using Generalized Linear Models. All analyses were conducted in R statistical software.

## Additional Information

**How to cite this article**: Chen, H. *et al.* Winter cropping in *Ficus tinctoria*: an alternative strategy. *Sci. Rep.* **5**, 16496; doi: 10.1038/srep16496 (2015).

## Figures and Tables

**Table 1:** The contents of the mature male syconia of *Ficus tinctoria* (mean ± SD), before the peak crop (Oct-Dec), during the peak crop (Jan-Feb), and after the peak crop (March).

Crops	N	Male flowers	Female flowers	Total flowers	Pollinators	Total wasps	NPFW (%)
Oct.-Dec.	90	12.94 ± 2.43	279.39 ± 68.06	292.33 ± 68.46	189.12 ± 69.03	190.43 ± 68.56	0.86 ± 2.62
Jan.-Feb.	240	12.41 ± 1.65	286.61 ± 49.86	299.02 ± 50.44	163.73 ± 54.42	178.36 ± 52.70	8.43 ± 11.19
March	30	11.80 ± 1.73	217.07 ± 56.54	228.87 ± 20.61	44.40 ± 23.69	102.37 ± 26.70	58.37 ± 14.46
